# 2-Deoxy-d-glucose Promotes Buforin IIb-Induced Cytotoxicity in Prostate Cancer DU145 Cells and Xenograft Tumors

**DOI:** 10.3390/molecules25235778

**Published:** 2020-12-07

**Authors:** Yangke Wanyan, Xixi Xu, Kehang Liu, Huidan Zhang, Junai Zhen, Rong Zhang, Jumei Wen, Ping Liu, Yuqing Chen

**Affiliations:** Jiangsu Province Key Laboratory for Molecular and Medical Biotechnology, Life Sciences College, Nanjing Normal University, Nanjing 210000, China; 181202070@njnu.edu.cn (Y.W.); 201202042@njnu.edu.cn (X.X.); 201202031@njnu.edu.cn (K.L.); 201201029@njnu.edu.cn (H.Z.); 191202090@njnu.edu.cn (J.Z.); 191202114@njnu.edu.cn (R.Z.); 191202034@njnu.edu.cn (J.W.); 08201@njnu.edu.cn (P.L.)

**Keywords:** buforin IIb, 2-Deoxy-d-glucose, mitochondria, glycolysis, prostate cancer

## Abstract

Inhibition of the glycolytic pathway is a critical strategy in anticancer therapy because of the role of aerobic glycolysis in cancer cells. The glycolytic inhibitor 2-Deoxy-d-glucose (2-DG) has shown potential in combination with other anticancer agents. Buforin IIb is an effective antimicrobial peptide (AMP) with broad-spectrum anticancer activity and selectivity. The efficacy of combination treatment with 2-DG and buforin IIb in prostate cancer remains unknown. Here, we tested the efficacy of buforin IIb as a mitochondria-targeting AMP in the androgen-independent human prostate cancer cell line DU145. Combining 2-DG with buforin IIb had a synergistic toxic effect on DU145 cells and mouse xenograft tumors. Combination treatment with 2-DG and buforin IIb caused stronger proliferation inhibition, greater G1 cell cycle arrest, and higher apoptosis than either treatment alone. Combination treatment dramatically decreased L-lactate production and intracellular ATP levels, indicating severe inhibition of glycolysis and ATP production. Flow cytometry and confocal laser scanning microscopy results indicate that 2-DG may increase buforin IIb uptake by DU145 cells, thereby increasing the mitochondria-targeting capacity of buforin IIb. This may partly explain the effect of combination treatment on enhancing buforin IIb-induced apoptosis. Consistently, 2-DG increased mitochondrial dysfunction and upregulated Bax/Bcl-2, promoting cytochrome c release to initiate procaspase 3 cleavage induced by buforin IIb. These results suggest that 2-DG sensitizes prostate cancer DU145 cells to buforin IIb. Moreover, combination treatment caused minimal hemolysis and cytotoxicity to normal WPMY-1 cells. Collectively, the current study demonstrates that dual targeting of glycolysis and mitochondria by 2-DG and buforin IIb may be an effective anticancer strategy for the treatment of some advanced prostate cancer.

## 1. Introduction

Despite a progressive decrease in death rate in the past two decades, cancer remains a major public health problem globally [1]. Prostate cancer (PCa) is the second most prevalent cancer and the fifth leading cause of cancer-related mortality in men globally [2]. The standard treatment for advanced PCa is based on hormonal strategies, such as androgen ablation therapy and/or androgen receptor (AR) antagonists, which are used to prevent AR signaling associated with the development and progression of PCa [3]. However, despite a good initial response, most patients eventually develop lethal castration-resistant PCa [4]. Current treatments are only slightly effective in such patients. Although several chemotherapeutic drugs, such as docetaxel and cabazitaxel, are approved for the treatment of advanced PCa, their efficacy is limited. Docetaxel is frequently associated with hematological toxicities such as neutropenia, and the use of mitoxantrone is limited by severe adverse events [5]. Hence, new treatment strategies against PCa are needed.

Approximately 250 natural and synthetic antimicrobial peptides (AMPs) with anticancer activities have been identified to date (http://aps.unmc.edu/AP/database/antiC.php), representing an attractive anticancer resource. In addition to their anticancer activity, some AMPs show specificity for cancer cells, suggesting that they could be alternative chemotherapeutic agents to overcome the limitations of current drugs [6]. Buforin IIb, an AMP derived from histone H2A, is an attractive candidate because it effective cytotoxicity against more than 60 cancer cell lines but low cytotoxicity to normal cells [7]. Buforin IIb selectively targets breast cancer cells by interacting with the sialic acids of glycoproteins or gangliosides on the cell surface [8]. A recent study showed that buforin IIb significantly inhibits proliferation and activates apoptosis in PCa cells [9]. However, high concentrations of buforin IIb are cytotoxic to normal human fibroblasts and erythrocytes [8,10]. Therefore, reducing the effective dose of buforin IIb is necessary to develop this peptide as a viable anticancer drug.

Preclinical evidence suggests that targeting the deregulated glucose metabolism is a potentially effective anticancer approach [11]. Unlike normal cells, cancer cells highly rely on glycolysis to obtain energy, even under aerobic conditions. Rather than mitochondrial oxidative phosphorylation, cancer cells derive energy from glycolysis even in the presence of normal oxygen concentrations in a process known as aerobic glycolysis or the Warburg effect. For this reason, targeting glucose metabolism could eliminate cancer cells selectively [12]. One of the most frequently used inhibitors of glucose metabolism is 2-Deoxy-d-glucose (2-DG), which is phosphorylated to 2-DG-P by hexokinase. 2-DG-P accumulates in the cell and inhibits hexokinase activity, thereby blocking glycolysis, acting in multiple cellular pathways to suppress tumor growth [13]. Jeon et al. (2015) demonstrated that 2-DG has anticancer activity in PCa cells by inhibiting cell proliferation, although its effect on causing cell death is limited [14]. Furthermore, high concentrations of 2-DG are needed to inhibit the glycolytic metabolism of cancer cells, and animal and clinical trials show that high doses of 2-DG cause adverse reactions [13,15,16]. These issues limit the therapeutic application of 2-DG in cancer. Several studies have reported that 2-DG sensitizes cancer cells to other anticancer molecules, indicating that the efficacy of some anticancer agents may improve when combined with 2-DG [17,18]. However, to date, few studies have examined the anticancer effect of combination treatment with 2-DG and AMPs.

Here, we aimed to determine whether 2-DG could sensitize human PCa androgen-independent DU145 cells to buforin IIb in vitro and in vivo. The results showed that 2-DG synergistically increased the anticancer activity of buforin IIb in DU145 cells and DU145 xenograft mice tumors. Combination treatment with 2-DG and buforin IIb had a stronger inhibitory effect on cell viability and inducing apoptosis than each agent alone. Our results suggest that inhibition of glycolysis by 2-DG is a promising agent to increase the therapeutic efficacy of AMPs buforin IIb in prostate cancer.

## 2. Results

### 2.1. Combination Treatment with 2-DG and Buforin IIb Synergistically Inhibits DU145 Cell Viability

**Figure 1:**
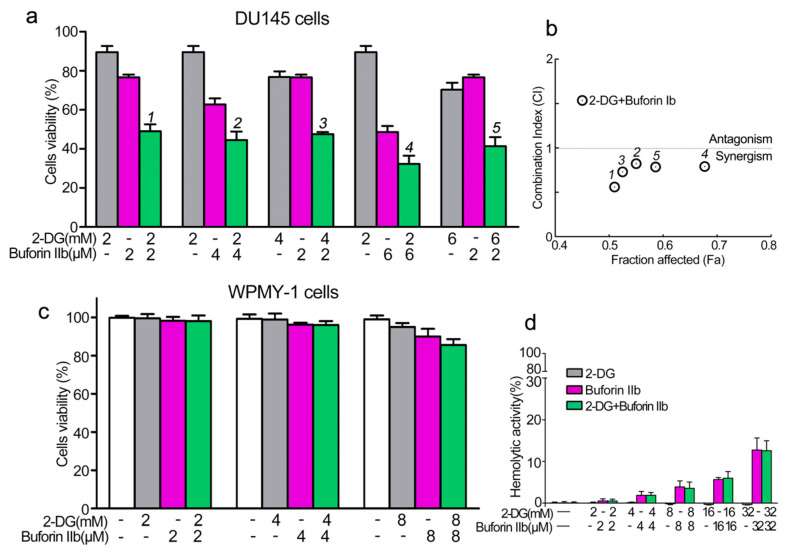
Effects of 2-DG and buforin IIb alone or in combination on prostate cancer and normal cells. (**a**) Cell viability in the prostate cancer cell line DU145 was assessed using the CCK-8 assay following 2-DG and/or buforin IIb treatment for 24 h. (**b**) The combination index (CI) of treatment with 2-DG and buforin IIb was measured by Compusyn software. (**c**) Cell viability in the normal prostatic epithelial WPMY-1 cell line. (**d**) Hemolytic activity was tested in mouse erythrocytes. The results are expressed as the mean ±SEM of 4–6 independent experiments.

PCa cell line DU145 and the normal prostate epithelial cell line WPMY-1 were used to assess the effect of combination treatment with 2-DG and buforin IIb on cell viability using the CCK8 assay. Buforin IIb and 2-DG inhibited DU145 cell viability in a dose-dependent manner, with IC_50_ values of 6 μM for Buforin IIb and 12 mM for 2-DG (data not shown). The inhibitory effects of the two agents in combination were assessed against DU145 at different concentrations (Figure 1a,b). All combination treatments showed greater inhibition of viability against DU145 cells than 2-DG and buforin IIb treatment alone. The combination index (CI) allows the quantitative determination of drug interactions, where CI < 1, = 1, and >1 indicates synergism, additive effect, and antagonism, respectively. Treatment with the two agents resulted in CI < 1 for all tested concentrations in DU145 cells, indicating that the combination of 2-DG and buforin IIb had a synergistic effect in DU145 cells. Combination treatment with 2 mM 2-DG and 2 μM buforin IIb had the lowest CI (CI = 0.56) on DU145 cell viability, indicating this combination had the strongest synergistic effect. Under this combination, the inhibition of DU145 cell viability induced by 2 μM buforin IIb alone increased from 25% to 50%. Thus, subsequent experiments were conducted using this combination in DU145 cells.

The cytotoxicity of combination treatment at different concentrations was detected in normal prostate epithelial WPMY-1 cells. No significant inhibition of viability of WPMY-1 cells was observed after treatment with 4 mM 2-DG and 4 μM buforin IIb alone or in combination (Figure 1c). In addition, 2-DG showed no effect on the viability of WPMY-1 cells at all tested concentrations. Also, buforin IIb showed very low cytotoxicity against WPMY-1 cells at the concentration of 8 μM. Hemolytic assays showed that 2-DG had no hemolytic activity (Figure 1d), whereas buforin IIb caused a certain amount of hemolysis. Combination treatment with 2 mM 2-DG and 2 μM buforin IIb did not cause hemolysis, whereas 32 mM 2-DG and 32 μM buforin IIb caused approximately 15% hemolysis. Thus, 2-DG did not affect the hemolytic effect of buforin IIb. These data indicate that 2-DG and buforin IIb synergistically and selectively inhibited the viability of DU145 cells.

### 2.2. Combination Treatment with 2-DG and Buforin IIb Synergistically Inhibits DU145 Cell Proliferation and Induces G1 Cell Cycle Arrest

**Figure 2:**
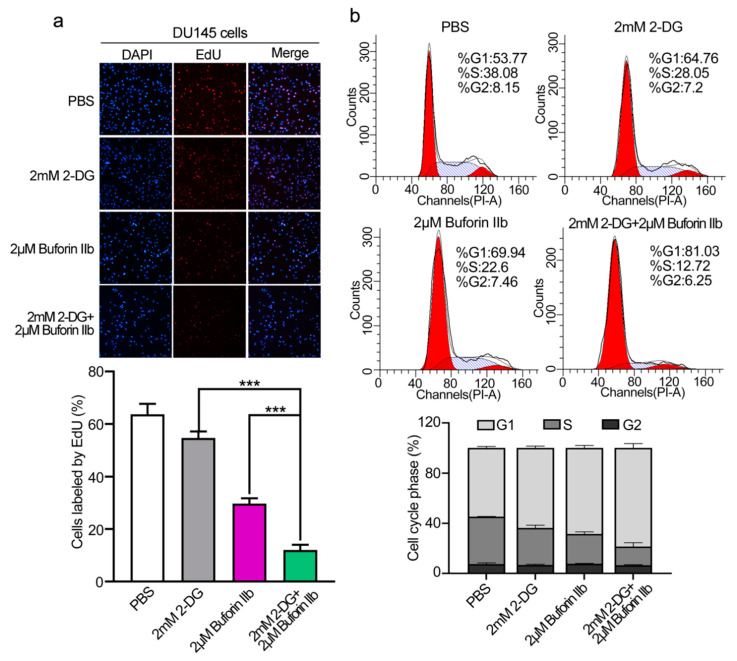
Effects of 2-DG and buforin IIb alone or in combination on cell proliferation and cell cycle progression in DU145 cells. (**a**) Cell proliferation was assessed using the EdU incorporation assay after treatment with 2-DG (2 mM) and buforin IIb (2 μM) alone or in combination for 24 h. Cells were visualized using a fluorescence microscope equipped with a filter for Ex/Em = 555/565 nm. *** *p* < 0.001. (**b**) Propidium iodide (PI) staining was performed to assess the cell cycle by FACS analysis.

The EdU assay was performed to assess DU145 cell proliferation. 2-DG and buforin IIb inhibited cell proliferation at a rate of 14% and 53%, respectively (Figure 2a). Combination treatment with 2 μM buforin IIb and 2 mM 2-DG significantly inhibited cell proliferation at a rate of 81% (*p* < 0.001). Flow cytometry showed that the G0/G1 population in DU145 cells increased from 53.7% in the PBS control to 64.76%, 69.94%, and 81.03%, following 2-DG and buforin IIb alone or in combination, respectively (Figure 2b). Accordingly, the S/G2 population decreased from 46.23% to 35.25%, 30.06%, and 18.97% following 2-DG and buforin IIb alone or combination treatment, respectively. Thus, combination treatment significant inhibited cell proliferation and induced cell cycle arrest at G1 phase in DU145 cells.

### 2.3. Combination Treatment with Buforin IIb and 2-DG Significantly Induces Apoptosis and Metabolic Dysfunction in DU145 Cells

**Figure 3:**
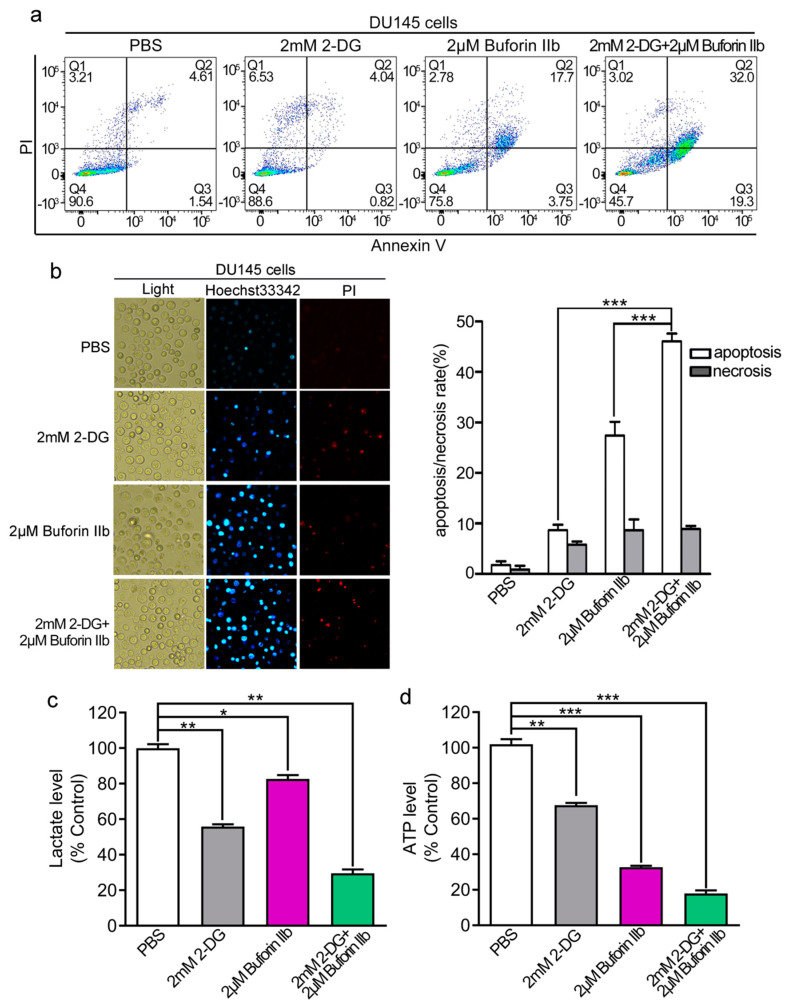
Apoptosis and metabolic levels in DU145 cells treated with buforin IIb or 2-DG alone or in combination. DU145 cells were treated with buforin IIb or 2-DG alone or in combination, stained with Annexin V-FITC/PI, and analyzed by flow cytometry (**a**) or stained by Hoechst 33342/PI and then observed by fluorescence microscopy (**b**). Lactate levels in the medium (**c**) and intracellular ATP levels (**d**) were measured after treatment with 2-DG alone for 24 h, buforin IIb alone for 12 h, or pretreatment with 2-DG for 12 h followed by buforin IIb for combination treatment for 12 h. * *p* < 0.05, ** *p* < 0.01, *** *p* < 0.001.

Buforin IIb induces apoptosis in several cancer cell lines [7]. Here, we examined the effect of 2-DG and buforin IIb on apoptosis in DU145 cells by Annexin V/PI staining and FACS analysis. As shown in Figure 3a, 2-DG alone did not induce DU145 cell apoptosis compared with the PBS control. Combination treatment with 2 mM 2-DG and 2 μM buforin IIb increased apoptosis (51.3%) compared with the effect of 2 μM buforin IIb alone (21.45%). Hoechst 33342/PI staining showed that the number of Hoechst 33342 positive cells was significantly higher in samples treated with 2-DG and buforin IIb in combination than in those treated with 2-DG or buforin IIb alone (*p* < 0.001) (Figure 3b). These data suggest that 2-DG significantly increased buforin IIb-induced apoptosis in DU145 cells.

Compared with 12 h treatment, 2-DG exerted stronger cytotoxicity to DU145 cells after 24 h treatment (Supplementary Figure S1). Considering the metabolic dysfunction, mitochondrial dysfunction, and early apoptosis signal usually occurs within 12 h induced by AMPs [8,19], pretreatment with 2-DG for 12 h and then adding buforin IIb was designed to evaluate the effect of combination treatment in the following study. The effect of combination treatment on L-lactate and ATP levels was assessed next. Treatment with 2 mM 2-DG or 2 μM buforin IIb alone significantly decreased lactate production to approximately 60% and 80% of that in the control, respectively. However, combination treatment with buforin IIb and 2-DG decreased L-lactate production to <30% of that in the control (Figure 3c). ATP levels significantly decreased to 70% and 30% of control levels in DU145 cells treated with 2 mM 2-DG and 2 μM buforin IIb alone, respectively. When 2-DG was combined with buforin IIb, intracellular ATP levels dropped >80% (Figure 3d). Therefore, combination treatment decreased L-lactate production and ATP to significantly lower levels than buforin IIb and 2-DG alone, indicating combination treatment with buforin IIb dramatically enhanced the effect of 2-DG on decreasing L-lactate production and intracellular ATP levels.

### 2.4. 2-DG Sensitizes DU145 Cells and Increases the Uptake of Buforin IIb

**Figure 4:**
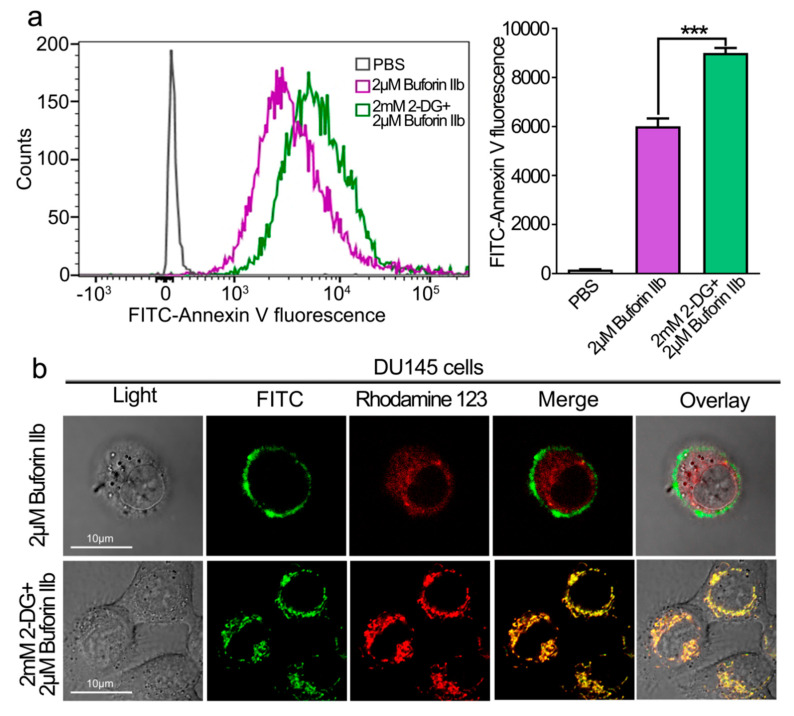
Effect of 2-DG on cellular entry and mitochondrial binding of FITC -buforin IIb in DU145 cells. (**a**) DU145 cells pretreated with or without 2-DG for 24 h were incubated with FITC-buforin IIb for 2 h at 37 °C, and fluorescence was detected by flow cytometry. Results are expressed as the mean ± SEM of three independent experiments. Relative membrane affinities were calculated and expressed in a bar chart. *** *p* < 0.001. (**b**) DU145 cells pretreated with or without 2-DG for 24 h were stained with rhodamine 123 for 30 min, followed by incubation with FITC-buforin IIb for 2 h, and then observed by confocal microscopy.

The effect of 2-DG on the cellular uptake of buforin IIb was examined by FACS analysis (Figure 4a). DU145 cells were pretreated with 2-DG for 24 h followed by FITC labeled buforin IIb. FITC fluorescence intensity was significantly higher in DU145 cells pretreated with 2-DG than in those treated with buforin IIb alone (*p* < 0.001). The intracellular distribution and localization of FITC-buforin IIb was detected using rhodamine 123 (a dye for mitochondria) and laser scanning confocal microscopy (Figure 4b). After treatment with FITC-labeled buforin IIb alone for 2 h, FITC fluorescence was mainly distributed on the surface of DU145 cells. However, cells pretreated with 2-DG showed increased FITC fluorescence, especially in mitochondria. This indicated that 2-DG may sensitize DU145 cells to the cellular uptake of buforin IIb, thus enhancing the accumulation of buforin IIb in mitochondria.

### 2.5. 2-DG Significantly Enhances Buforin IIb-Induced Mitochondrial Dysfunction

**Figure 5:**
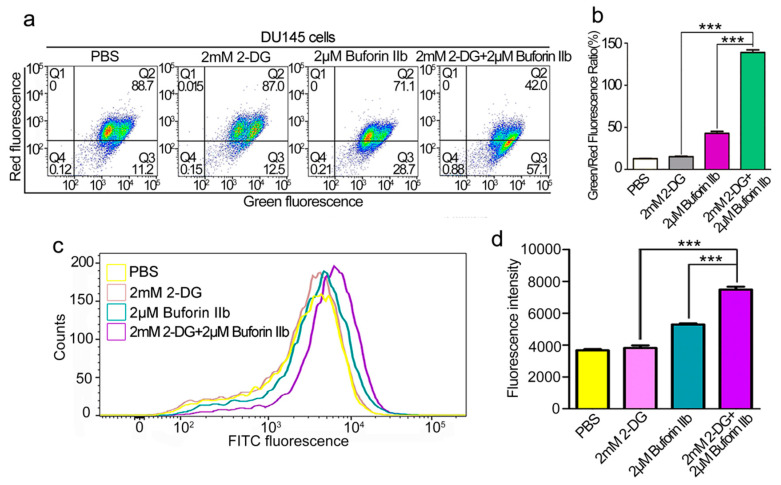
Effects of 2-DG and buforin IIb alone or in combination on mitochondria in DU145 cells. (**a**) DU145 cells pretreated with or without 2-DG (2 mM) for 12 h were incubated with buforin IIb (2 μM) for 12 h, followed by staining with JC-1 and flow cytometry at 490 nm and 525 nm excitation. The emitted light was detected at 590 nm (red) and 530 nm (green). (**b**) The mitochondrial membrane potential was expressed as the ratio of green to red fluorescence intensity. (**c**) DU145 cells pretreated with or without 2-DG (2 mM) for 12 h were incubated with buforin IIb (2 μM) for 6 h, and then stained with H2DCFDA for 30 min. Cells were analyzed by flow cytometry at 488 nm excitation. (**d**) Statistical analysis of fluorescence intensity from (**c**). *** *p* < 0.001.

The effect of combination treatment on the mitochondrial membrane potential (MMP, Δψm) was assessed using the JC-1 fluorescent probe. As shown in Figure 5a,b, 2 mM 2-DG had no effect on Δψm in DU145 cells. Treatment with 2 μM buforin IIb increased the green/red fluorescence ratio in DU145 cells, indicating that buforin IIb had an effect on Δψm. Combination treatment increased the green/red fluorescent ratio by >3-fold over that of 2 μM buforin IIb alone. This indicated that combination treatment caused a stronger disruption of the Δψm. Quantification of ROS in DU145 cells using the H2DCFDA fluorescent dye showed that 2-DG had no effect on H2DCFDA fluorescence intensity (Figure 5c,d). Combination treatment with buforin IIb and 2-DG significantly increased fluorescence intensity, compared with 2 μM buforin alone (*p* < 0.001). Thus, combination treatment significantly increased ROS levels. These data indicate that 2-DG significantly increased mitochondrial dysfunction induced by buforin IIb.

### 2.6. 2-DG Promotes Buforin IIb-Induced Mitochondria-Dependent Apoptosis in DU145 Cells

**Figure 6:**
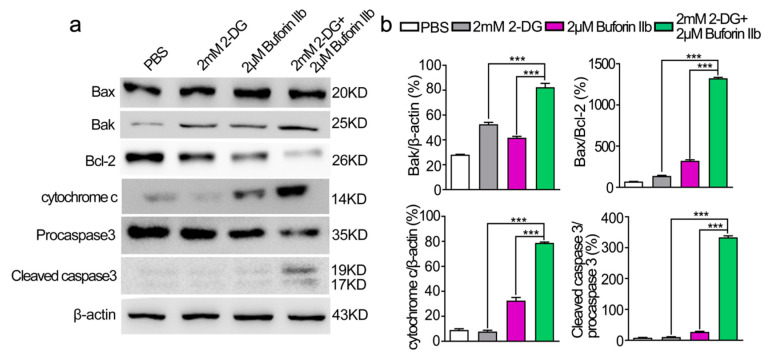
Effects of 2-DG and buforin IIb alone or in combination on mitochondria-dependent apoptosis in DU145 cells. (**a**) Cells pretreated with or without 2-DG (2 mM) for 12 h were incubated with buforin IIb (2 μM) for 12 h. The lysates were harvested and apoptosis-related proteins were detected by western blotting. (**b**) The relative amounts of Bak, Bax/Bcl-2, cytochrome c, cleaved caspase 3/procaspase 3 were determined by western blotting and Image J densitometric analysis. Results are the mean ± SEM of three independent experiments. *** *p* < 0.001.

To determine whether combination treatment induced mitochondria-dependent apoptosis, several proteins involved in the mitochondria-dependent apoptosis pathway were detected in DU145 cells treated with 2-DG and/or buforin IIb (Figure 6). Assessment of the expression of anti-apoptotic Bcl-2 and pro-apoptotic Bax and Bak in DU145 cells showed that 2-DG and buforin IIb downregulated Bcl-2 and upregulated Bak. Combination treatment with 2-DG and buforin IIb significantly decreased the level of Bcl-2 and increased the Bax/Bcl-2 ratio (*p* < 0.001). Buforin IIb increased cytochrome c levels and induced the cleavage of procaspase 3 in DU145 cells, whereas 2-DG alone did not promote the release of cytochrome c from mitochondria or activate caspase 3. However, combination treatment significantly increased the levels of cytochrome c and cleaved caspase 3 (*p* < 0.001). These results indicate that 2-DG significantly increased buforin IIb-induced activation of mitochondrial apoptosis in DU145 cells.

### 2.7. Combination Treatment Showed Signifcant Anti-Tumor Efects in DU145 Xenograft Tumors

**Figure 7:**
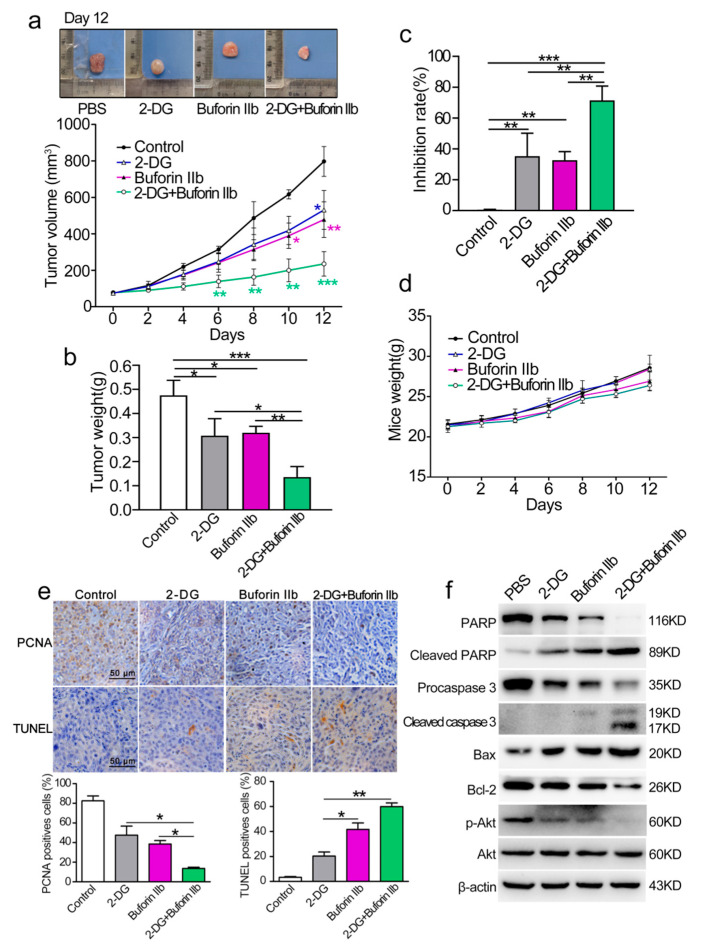
Effect of 2-DG combined with buforin IIb on DU145 xenograft tumors. (**a**) Tumor growth curves over time. The difference comparison was conducted between different treatment groups and PBS control group. (**b**) On day 12, tumors were carefully excised and tumor weight was measured. (**c**) Comparison of inhibition rate of tumor growth in different treatment groups. (**d**) Weight curves of mice over time. (**e**) Paraffin sections were examined by immunohistochemical staining using anti-PCNA antibody and TUNEL staining. Representative images from each group are shown. (**f**) Tumor tissues were crushed and lysed with RIPA lysis buffer, and the supernatants were collected for western blot analysis using the indicated antibodies. Similar results were obtained in at least three independent experiments. * *p* < 0.05, ** *p* < 0.01, *** *p* < 0.001.

The anticancer effect of combination treatment with buforin IIb and 2-DG was assessed in a DU145 xenograft nude mouse model. BALB/c nude mice were subcutaneously injected with DU145 cells and then randomly divided into four groups as follows: PBS (control), buforin IIb (5 mg/kg), 2-DG (400 mg/kg), and combination group (5 mg/kg buforin IIb + 400 mg/kg 2-DG). The tumor volume of DU145 tumor xenograft receiving 2-DG, buforin-IIb, and combination treatment was lower than that of the PBS control mice (Figure 7a). Further analysis showed that tumor volume and tumor weight were significantly lower in the combination treatment group than in the buforin IIb or 2-DG alone groups (Figure 7a,b). Combination treatment significantly suppressed tumor growth compared with either 2-DG or buforin IIb alone. The tumor growth inhibition rate of 2-DG, buforin IIb, and combination groups was 39.53%, 34.32%, and 74.78%, respectively, compared with the control group (Figure 7c). The body weight of mice did not differ significantly among the groups (Figure 7d). These data suggest that the combination of 2-DG and buforin IIb is more effective for inhibiting the growth of DU145 xenograft tumors than 2-DG or buforin IIb alone.

PCNA and TUNEL immunohistochemical were used to quantify cell proliferation and apoptosis in tumor sections from all groups. Comparing with the buforin IIb or 2-DG alone groups, significantly decreased PCNA positive cells and increased TUNEL positive cells were detected in the combination treatment group (Figure 7e). The results of western blot analysis of tumor protein lysates are shown in Figure 7f. The levels of Bax, cleaved caspase 3, and cleaved PARP were significantly higher, whereas the level of Bcl-2 was significantly lower in tumor tissues of mice receiving combination treatment than in those receiving 2-DG or buforin IIb alone, indicating that combination treatment induced stronger mitochondria-dependent apoptosis. Therefore, the combination of 2-DG and buforin IIb in vivo causes an effective tumor regression through the induction of mitochondria-dependent apoptosis in DU145 xenografts in mice.

## 3. Discussion

Current treatment strategies for advanced PCa lack selectivity and efficacy, underscoring the need to identify effective and selective anticancer agents for the treatment of advanced PCa. In recent years, AMPs have attracted attention as effective anticancer drugs. In addition to their common membranolytic mode of action via a non-receptor-mediated pathway, several anticancer AMPs can cross the membrane and access the intracellular compartment to target mitochondria, thereby inducing programmed cell death in cancer cells [19,20,21]. Buforin IIb is an effective AMP because of its broad-spectrum anticancer activity. It induces apoptosis in human cervical carcinoma HeLa cells by affecting ER stress-mediated mitochondrial membrane permeabilization [22]. Buforin IIb suppresses the progression of liver cancer by inducing cell apoptosis and inhibiting cell viability and cell migration [23]. In previous work from our group, we demonstrated the specificity of buforin IIb by showing that it binds strongly to sialylated oligosaccharides of glycoproteins and glycolipids on the surface of breast cancer cells, leading to its uptake into cells and the induction of mitochondria-dependent apoptosis [8]. Buforin IIb induces apoptosis of PC-3 and DU145 cells by modulating the caspase pathway and p53-Bcl-2 family proteins [9]. In the current study, we demonstrated that buforin IIb targets mitochondria in DU145 cells. Buforin IIb affected mitochondrial function by increasing the Δψm and increasing ROS production and the release of cytochrome c from mitochondria, resulting in caspase-dependent apoptosis. Therefore, similar to most non-membranolytic AMPs, buforin IIb functions as a typical mitochondria-targeting anticancer AMP that directly affects mitochondrial activity. Mitochondria are the source of energy for cell metabolism, and functional mitochondria are essential for cancer cell viability. Mitochondria-targeting therapies have advanced the treatment of cancer, including targeting mitochondrial metabolism, ROS production, the permeability transition pore complex, and outer membrane permeabilization [24]. Here, we identified buforin IIb as a mitochondria-targeting anticancer AMP that caused mitochondria-dependent apoptosis of prostate cancer DU145 cells.

Glycolysis inhibitors have been studied extensively in recent years as a group of compounds with a potential anticancer activity. This is associated with increasing knowledge of metabolic disorders in cancer cells. One difference between cancer cells and normal cells is that the metabolism of cancer cells is reprogrammed to generate energy from aerobic glycolysis instead of oxidative phosphorylation, which affects mitochondrial function [25]. One effective anticancer strategy is to combine mitochondrial and antiglycolytic drugs with different molecular targets, which may elicit synergistic effects. 2-DG is a typical inhibitor of glycolysis that can interfere with D-glucose metabolism, supporting that nutrient and energy deprivation is an efficient strategy to suppress cancer cell growth and survival [16]. In addition to glycolysis inhibition, other biological effects such as autophagy/apoptosis induction and N-glycosylation inhibition were also explored [26,27]. The present study used a relatively low concentration of 2-DG (2 mM), and the results showed that 2 mM 2-DG did not inhibit DU145 cell growth significantly, and it had no effect on mitochondrial Δψm and ROS production. Sahra et al. (2010) reported that 1 mM 2-DG alone had almost no effect on cell viability and apoptosis in DU145 cells, which contain a p53 mutation, suggesting that functional p53 is required for the induction of apoptosis by 2-DG in PCa cells [28]. In our study, treatment with 2 mM 2-DG upregulated Bak and downregulated Bcl-2 levels; however, no cytochrome c release from mitochondria or pro-caspase 3 cleavage were observed in DU145 cells. Thus, 2 mM 2-DG did not have the ability to induce mitochondria-dependent apoptosis in DU145 cells.

The therapeutic efficacy of 2-DG as a single agent is limited, and it is often combined with other therapeutic agents to obtain a synergistic anticancer effect [16,29]. Here, we assessed the effect of the combination of AMPs and 2-DG for the first time. All tested combinations of 2-DG and buforin IIb at different concentrations had a synergistic effect in DU145 cells. Of these, combination with 2 mM 2-DG and 2 μM buforin IIb showed the strongest synergistic effect. However, combinations of 2-DG and buforin IIb had a little synergistic effect in prostate cancer PC-3 cells (Supplementary Figure S2). Thus, DU145 cells were more sensitive to combination treatment with 2-DG and buforin IIb. 2-DG alone at a dose of 2 mM had no significant effect on cell viability and apoptosis in DU145 cells. Buforin IIb at 2 μM caused a 25% decrease of cell viability in DU145 cells. However, combination treatment led to 50% cell death. The combination of 2 μM buforin IIb with 2 mM 2-DG thus achieved moderate synergistic effects on the inhibition of cell viability in DU145 cells. Further analysis revealed that the synergistic effects were the result of increased proliferation inhibition, G1 phase arrest, and apoptosis induction. Dual targeting of mitochondrial and glycolytic pathways was proposed as a promising chemotherapeutic strategy, and the combination of 2-DG and mitochondria-targeted anticancer agents generated a synergistic anticancer effect in previous studies [30,31]. A report showed that the inhibitor of mitochondrial oxidative phosphorylation oligomycin in combination with 2-DG can rapidly eradicate tumor cells. However, because of the lack of selectivity, the combination also increases the toxicity to normal cells [32]. The synergistic anticancer effect of the combination of 2-DG with metformin and mitochondria-targeted carboxy-proxyl (such as Mito-CP, Mito-Q, and Mito-CP-Ac) on inhibiting proliferation and migration and promoting apoptosis has been analyzed, which showed a certain selectivity for cancer cells [28,33,34]. However, few mitochondria-targeted anticancer molecules have been identified. In the present study, treatment with 2 mM 2-DG and 2 μM buforin IIb alone or in combination showed no significant cytotoxicity against WPMY-1 cells. The results of the hemolytic assay showed that 2 mM buforin IIb did not cause hemolysis. Thus, buforin IIb is an effective mitochondria-targeted selective anticancer candidate for PCa.

2-DG (2 mM) alone had no effect on mitochondrial Δψm and ROS production. However, 2 mM 2-DG combined with 2 μM buforin IIb caused a 3-fold increase of mitochondrial Δψm and a 30% increase in ROS levels over those caused by buforin IIb alone. This implied that 2-DG increases the mitochondrial dysfunction induced by buforin IIb through an unidentified pathway. A similar phenomenon was observed in response to combination treatment with 2-DG and metformin or sonodynamic therapy in breast cancer cells [35,36]. FACS analysis showed that pretreatment with 2 mM 2-DG increased the binding affinity of buforin IIb to DU145 cells, as indicated by stronger FITC fluorescence. CLSM observation confirmed that the amount of FITC-buforin IIb on mitochondria was higher in 2-DG pretreated DU145 cells. This indicates that 2-DG may sensitize DU145 cells, thereby promoting the entry of buforin IIb into cells. Thus, the enhanced effect on mitochondrial dysfunction may be partly caused by increased accumulation in mitochondria induced by the combination of buforin IIb and 2-DG. We hypothesized that 2-DG may change cell conditions by inhibiting glycolysis and the generation of glycolytic intermediates that are precursors to the anabolic processes, thereby facilitating the transport of buforin IIb across the membrane in DU145 cells. Additional research is needed to clarify this issue.

Combination treatment increased ROS levels markedly and decreased Δψm considerably in DU145 cells, which may lead to increased mitochondrial membrane permeability. This is an important event resulting in the release of cytochrome c from the mitochondria into the cytosol, which leads to the activation of caspase and the induction of apoptosis [37]. Many AMPs have the ability to directly affect MOMP, resulting in the release of cytochrome c [20,38]. Here, the levels of cytochrome c increased by approximately 2-fold, and cleaved caspase 3 increased by >10-fold in response to the combination of 2 μM buforin IIb and 2 mM 2-DG compared with 2 μM buforin IIb alone. The present data are consistent with a previous report showing that 2-DG alone downregulates Bcl-2 [39]. In addition, the present data indicate that 2-DG upregulated Bak, whereas it did not cause the release of cytochrome c. This suggests that 2-DG does not affect MOMP by regulating Bak/Bcl-2. Bcl-2 family proteins play crucial roles in the intrinsic pathway by regulating the release of cytochrome c from mitochondria, resulting in the activation of executioner caspases. Targeting the Bcl-2 family of anti-apoptotic proteins is an effective strategy for apoptosis induction in several cancer cells [40]. The pro-apoptotic proteins Bax and Bak are present in 95–100% and 77.5%, respectively, of PCs tissues evaluated regardless of tumor grade [41]. In both DU145 cells and DU145 xenograft tumors, the combination of buforin IIb and 2-DG downregulated Bcl-2 and upregulated Bax/Bcl-2 significantly, as well as upregulating the proapoptotic protein Bak. This suggests that 2-DG affected the mitochondrial apoptosis-related proteins Bcl-2, Bax, and Bak, which contributed to buforin IIb effect, and led to MOMP change in DU145 cells. This increased the release of cytochrome c and activated caspase-dependent apoptosis. Thus, developing the buforin IIb and 2-DG combination as a strategy for targeting Bcl-2 family proteins and inducing apoptosis would be effective for the treatment of androgen-independent PCa.

As an inhibitor of glycolysis, 2-DG decreased ATP generation and L-lactate production in DU145 cells. Buforin IIb also decreased ATP levels significantly in DU145 cells. This may be associated with the dysfunction of mitochondria, as indicated by increased ROS levels and decreased Δψm in DU145 cells. 2-DG and buforin IIb inhibited glycolysis and respiration and led to severe depletion of intracellular ATP, which would serve as an important mechanism for the synergistic anticancer effect. Several reports indicate that combination with 2-DG increases the effect of agents on decreasing ATP levels [42,43]. Combination treatment of 2-DG with buforin IIb also decreased L-lactate production. Thus, combination treatment led to metabolic dysfunction in DU145 cells, which may contribute partly to the enhanced anticancer activity.

Cell surface binding and internalization are critical for the specific targeting and biofunction of some cationic AMPs with anticancer activity. Buforin IIb has selective anticancer activity. The selectivity is associated with a higher binding ability to cancer cells than to normal cells, resulting in enhanced uptake into cancer cells [8]. Tumor cells have a more negative mitochondrial transmembrane potential than normal cells [44], which may drive the mitochondrial accumulation of the cationic buforin IIb. Treatment with 2-DG and buforin IIb alone or in combination did not inhibit the viability of normal prostate epithelial WPMY-1 cells. The results of the hemolytic assay showed that 2-DG had no effect on the hemolytic activity of buforin IIb, although buforin IIb caused hemolysis at high concentrations. Combination treatment effectively decreased the therapeutic dose of both buforin IIb and 2-DG, and thus would be sufficient to eradicate cancer cells with minimal side effects.

## 4. Materials and Methods

### 4.1. Reagents

Buforin IIb and fluorescein isothiocyanate (FITC)-buforin IIb were synthesized using solid-phase Fmoc methods by Synpeptide, Inc (Nanjing, China). The synthesized peptides were all >95% homogeneous as indicated by C18 reverse-phase HPLC and electrospray ionization (ESI) mass spectroscopy analysis.

RPMI-1640 was purchased from Wisent Inc (St-Bruno, QC, Canada), FBS from TransGen Biotech Co., Ltd., (Beijing, China). CCK-8 kit was purchased from Vazyme, Inc (Nanjing, China). 2-Deoxy-d-glucose was purchased from Aladdin (Shanghai, China). JC-1 Detection kit, DAPI solution, and H2DCFDA were purchased from KeyGEN BioTECH, Inc (Nanjing, China). Rhodamine 123 was purchased from Sigma-Aldrich (St. Louis, MO, USA). ATP content assay kit and hematoxylin solution was purchased from Solarbio Science and Technology (Beijing, China). L-lactate assay kit were purchased from Jiancheng Bioengineering Institute (Nanjing, China). ROS assay kit, Hoechst 33342/PI staining kit, Annexin V/PI assay kit, BeyoClick™ EdU Cell Proliferation Kit with Alexa Fluor 555 Cell Cycle Analysis Kit and 3,3′-Diaminobenzidine tetrahydrochloride (DAB) Horseradish Peroxidase Color Development Kit was purchased from Beyotime Institute of Biotechnology (Shanghai, China). Chemistar High-sig ECL Western Blotting Substrate was purchased from Tanon (Shanghai, China). Antibodies against PARP (#9532S), Bax (#5023S), Bcl-2 (#2870S), cytochrome c (#11940S), caspase 3 (#9662S), p-Akt (#4060S), Akt (#9272S), and PCNA(#13110) were purchased from CST, Inc. (CST, USA). β-Actin (AC026) was purchased from ABclonal (Wuhan, China). Bak (cat.no.sc-517390) was purchased from Santa Cruz Biotechnology (Santa Cruz, CA, United States). Biotinylated secondary antibody and streptavidin-biotin peroxidase complex were purchased from Boster Biological Technology (Wuhan, China). DAB (SA-HRP) TUNEL Cell Apoptosis Detection Kit was purchased from Servicebio (Wuhan, China). All other reagents were analytical grade reagents and produced in China. All the reagents were used by the rules of standard biosecurity and safety procedures of Nanjing Normal University.

### 4.2. Cell Lines and Cell Culture

The PCa cell lines DU145 and the normal prostatic epithelial cell line WPMY-1 were obtained from Shanghai Institute of Biochemistry and Cell Biology, Chinese Academy of Sciences. Cells were maintained in RPMI-1640 medium supplemented with 10% FBS, 100 μg/mL streptomycin, and 100 U/mL penicillin at 37 °C in a 5% CO_2_ humidified atmosphere.

### 4.3. Cell Viability Assay

Cells were seeded in 96-well plates at a density of 1 × 10^5^ cells/mL in medium, and then treated with different concentrations of buforin IIb and 2-DG alone or in combination for 24 h. After treatment, 10 μL CCK-8 was added to each well, and the plates were incubated in the dark at 37 °C for 2 h. Absorbance at 450 nm was measured using a SynergyTMH1 multifunction microplate reader. Each experiment was performed in triplicate, and cell viability was expressed as a percentage of the control. The drug–drug interactions between buforin IIb and 2-DG were evaluated using the combination index (CI) described by Chou and Talalay, and calculated using Compusyn software (Biosoft) [45]. CI values < 1, = 1 or > 1 indicated synergy, additivity, or antagonism, respectively.

### 4.4. Hemolytic Activity Assay

The hemolytic assay was conducted using mouse red blood cells (RBCs) as the method described previously [46]. Briefly, erythrocytes were isolated by centrifugation at 1000× *g* for 10 min, washed three times with PBS, and resuspended at a final concentration of 4% (*v/v*). After incubating cells with 2-DG and buforin IIb alone or in combination at different concentrations for 30 min at 37 °C, the mixture was centrifuged at 1000× *g* for 5 min to obtain the supernatant. A volume of 100 μL of supernatant was removed to a new 96-well plate and the absorbance was measured at 570 nm. PBS and 0.1% Triton X-100 were used as agents for 0 and 100% hemolysis, respectively. The percentage of hemolysis was calculated as follows: (A_sample_ − A_PBS_)/(A_TritonX-100_ − A_PBS_) × 100%, where A is the absorbance. Data are presented as the mean ± standard error of the mean (SEM) of at 4–6 independent experiments.

### 4.5. Cell Cycle Analysis

Cell cycle analysis was performed using the Cell Cycle Analysis Kit. Briefly, cells were seeded into 6-well culture plates at a density of 3 × 10^5^ cells/mL. After treatment with 2-DG (2 mM) and buforin IIb (2 μM) alone or in combination for 24 h, cells were digested by 0.25% trypsin without EDTA, fixed in 70% cold ethanol for 5 h at 4 °C, and then stained with propidium iodide (PI) and RNase A solution for 30 min at 37 °C in the dark. The cell distribution across the cell cycle was analyzed with a FACS Vantage SE flow cytometer.

### 4.6. 5-Ethynyl-20-Deoxyuridine (EdU) Incorporation Assay

Cultured cells were plated in 12-well plates at a density of 2 × 10^5^ cells/mL and treated with buforin IIb (2 μM) and 2-DG (2 mM) alone and in combination for 24 h. Cells were then incubated with in medium containing EdU (10 μM) for 2 h, fixed with 4% paraformaldehyde for 15 min, and permeabilized with 0.2% Triton X-100 for 15 min. Cells were then incubated with EdU staining cocktail at room temperature in the dark for 30 min. After washing with PBS, cell nuclei were stained with DAPI at a concentration of 10 μg/mL for 15 min. More than five images of each well were acquired by fluorescence microscope, and the percentage of EdU-positive cells was evaluated.

### 4.7. Hoechst 33342/PI Staining

Characteristic apoptotic morphological changes were assessed using the Hoechst 33342/PI staining kit. Briefly, DU145 cells were seeded (3 × 10^5^ cells/mL) into 6-well plates and treated with buforin IIb (2 μM) and 2-DG (2 mM) alone and in combination. After incubation for 24 h, apoptotic nuclei were stained by Hoechst 33342 (v/v at 1:100) and PI (v/v at 1:100) at 37 °C for 15 min in the dark, and then cells were washed three times by PBS. The images were obtained by fluorescence microscopy.

### 4.8. Annexin V/PI Staining

DU145 cells were seeded (3 × 10^5^ cells/mL) into 6-well plates and treated with buforin IIb (2 μM) and 2-DG (2 mM) alone and in combination for 24 h. After collecting and washing with PBS, the cells were re-suspended in 500 μL of binding buffer, stained with FITC-conjugated Annexin V and PI, and incubated on ice in the dark for 10 min. The stained cells were analyzed by flow cytometry, and the percentage of apoptotic cells was calculated using Cell Quest software.

### 4.9. Analysis of Colocalization with Mitochondria

Cells were seeded in a small confocal laser dish at 3 × 10^5^ cells/mL. For the combination group, cells were pretreated with 2-DG (2 mM) for 24 h, then incubated with 30 nM rhodamine 123 for 30 min in the dark. After harvesting, washing three times with PBS, cells were maintained in RPMI-1640 medium containing 2 μM FITC-buforin IIb at 37 °C for 2 h in the dark. The cells were washed three times with PBS, followed by observing using confocal laser scanning microscopy (CLSM) at 488 nm excitation and 525 nm emission wavelengths for FITC and 507 nm excitation and 529 nm emission wavelengths for rhodamine 123 signal detection.

### 4.10. Analysis the Cellular Binding of Buforin IIb by Flow Cytometry

Cells (3 × 10^5^ cells/mL) were seeded into 6-well plates. For the combination group, cells were pretreated with 2-DG (2 mM). After 24 h, cells were collected and resuspended in PBS. The binding activities of the buforin IIb were assessed after induction with 2 μM FITC-buforin IIb at 37 °C for 2 h in the dark. Finally, the cells were washed with PBS and analyzed with a FACS Vantage SE flow cytometer. The mean fluorescence of 10,000 cells was analyzed for each sample using BD flow cytometry software. Data are reported as the mean ± SEM of three independent experiments.

### 4.11. Measurement of Mitochondrial Membrane Potential

The JC-1 Assay Kit was used to measure changes in mitochondrial membrane potential. Briefly, cells (5 × 10^5^ cells/mL) were seeded into 6-well plates and treated with 2-DG (2 mM) for 12 h, then treated with or without buforin IIb (2 μM) for 12 h. DU145 cells were collected, washed in PBS, and incubated with 10 μg/mL JC-1 for 30 min. The cells were then collected by centrifugation and analyzed by flow cytometry. The red fluorescence indicated JC-1 aggregates, whereas green fluorescence indicated JC-1 monomers. Aggregated JC-1 (red fluorescence) indicates a high membrane potential, whereas monomer JC-1 (green fluorescence) indicates a membrane collapse.

### 4.12. Reactive Oxygen Species (ROS) Assay

Intracellular ROS level was measured by detecting the fluorescence intensity of the peroxide sensitive fluorescent probe H2DCFDA. Briefly, DU145 cells (1 × 10^5^ cells/mL) were seeded into 24-well plates and treated with 2-DG (2 mM) for 12 h, then treated with or without buforin IIb (2 μM) for 6 h, followed by incubation with 10 μM H2DCFDA for 30 min in the dark. The fluorescence intensity was measured at 488 nm by flow cytometry to evaluate the production of ROS.

### 4.13. Measurement of Lactate and ATP Levels

Cells were seeded at approximately 3 × 10^5^ cells/mL in 6-well plates and treated with 2-DG (2 mM) alone for 24 h and buforin IIb alone (2 μM) or both for 12 h. After centrifugation, the culture medium and cells were collected to estimate the L-lactate and ATP content using the ATP content assay kit (Solarbio, Beijing, China) and L-lactate assay kit (Jiancheng Bioengineering, Nanjing, China) according to the manufacturer’s protocol. Lactate dehydrogenase catalyzes the conversion of lactate to pyruvic acid, and the absorbance at 530 nm was determined spectrophotometrically after the addition of a color developing agent, which showed a linear relationship with lactate content. The collected cells were subjected to ultrasonic disruption and centrifuged at 8000× *g* for 10 min at 4 °C to collect the supernatant for intracellular ATP detection. Creatine kinase catalyzes the production of phosphocreatine from creatine and ATP. The level of ATP was determined by detecting the phosphocreatine content though the colorimetric method of phosphomolybdic acid at 700 nm. Six replicates were performed for each group, and the experiments were repeated three times to confirm the results.

### 4.14. Western Blot Analysis

DU145 cells were treated with 2-DG (2 mM) for 12 h, then treated with or without buforin IIb (2 μM) for 12 h. The cells were lysed on ice for 30 min in RIPA lysis buffer, and the lysates were centrifuged at 12,000× *g* for 10 min at 4 °C. The supernatant was collected, separated by 12% sodium dodecyl sulfate-polyacrylamide gel electrophoresis (SDS-PAGE), and transferred onto a polyvinylidene difluoride (PVDF) membrane. After blocking with 5% skim milk in PBS, the PVDF membrane incubated with primary antibodies against Bcl-2, Bak, Bax, cytochrome C, caspase 3, and β-actin overnight at 4 °C, followed by incubating with appropriate secondary antibodies. After washing, bands were visualized by using ECL detection system according to the manufacturer’s instructions.

### 4.15. Xenograft Tumor Experiment

Male BALB/c nude mice (4–6 weeks old) were obtained from the Model Animal Research Center of Nanjing University. All of the animal experiments were approved by the Institutional Animal Care and Use Committee (IACUC) of the Nanjing Normal University and Jiangsu Association for Laboratory Animal Science. BALB/c nude mice were subcutaneously injected with 3 × 10^6^ DU145 cells in the right axillary region. When the tumor volume reached 75 mm^3^, nude mice were randomly divided into the following four groups (n = 5 per group): PBS, buforin IIb (5 mg/kg), 2-DG (400 mg/kg), and combination group (5 mg/kg buforin IIb + 400 mg/kg 2-DG). Buforin IIb was injected through the tail vein of mice and 2-DG was administered via oral gavage on days 1, 2, 3, 6, and 9. Body weight and tumor volume were measured three times a week. Mice were sacrificed by isoflurane inhalation followed by cervical dislocation. The tumors were excised and assessed. Tumor volume was calculated using the formula *V* = ab^2^/2, where ‘a’ and ‘b’ are the tumor dimensions at the longest and widest points, respectively [43]. On day 12, tumor tissues were collected and weighed. The rate of inhibition of tumor growth was calculated from the tumor weight (TW, g) as Tumor growth inhibition rate = (TW_PBS_ − TW_Treatment_)/TW_PBS_ × 100%. Xenograft tumor tissue samples were weighed and incubated in RIPA lysis buffer (150 μL/20 mg) to prepare homogenates using tissue homogenizer. The lysates were centrifuged to collect the supernatant, which was analyzed by western blotting as described above. Primary antibodies against Bcl-2, Bax, procaspase 3, caspase 3, PARP, cleaved PARP, p-Akt, Akt, and β-actin were detected here.

### 4.16. Immunohistochemical Analysis for PCNA and TUNEL

Immunohistochemical staining of tumor specimens was performed as previously described [19]. The sections were stained using primary anti-PCNA antibody (1:200 dilution) overnight at 4 °C and then incubated with a biotinylated secondary antibody for 30 min. Tumor specimens were also examined by DAB (SA-HRP) TUNEL Cell Apoptosis Detection Kit (Servicebio, Wuhan, China). Briefly, the slides were treated with proteinase K for 20 min at 37 °C and then incubated in with 3% H_2_O_2_ methanol solution for 20 min at room temperature. The sections incubating with 50 μL of a TdT incubation buffer for 1h at room temperature in the dark. After being incubated with the streptavidin-biotin peroxidase complex for 30 min, the sections were exposed to DAB solution and then stained with hematoxylin solution. PCNA and TUNEL positive cells were quantified by measuring pixels in 10 consecutive fields at 40× magnification. Positive cells were scored manually.

### 4.17. Statistical Analysis

All data expressed as mean ± SEM were representative of at least three independent experiments. Statistical analysis was performed using GraphPad Prism (GraphPad Prism 5.0, La Jolla, CA, USA). The statistical significance of in vitro and in vivo data was assessed by comparing mean values (± SD) using two-tailed Student’s *t*-test, and one-way ANOVA with Dunnett’s multiple comparison test were used for calculating the significance between different groups. Significance was assumed at *p* < 0.05 (*), *p* < 0.01 (**), and *p* < 0.001 (***).

## 5. Conclusions

In summary, the results presented here indicate that the combination of buforin IIb and 2-DG exerted synergistic and selective anticancer effects on human androgen-independent prostate cancer cell line DU145 in vitro and in vivo. The synergistic anticancer activity was associated with multiple mechanisms, including enhanced proliferation and apoptosis induction, G1 phase arrest, glycolysis inhibition, and intracellular ATP depletion. An interesting finding was that 2-DG may sensitize DU145 cells, thereby increasing the uptake of buforin IIb into cells and the mitochondria-targeting capability of buforin IIb alone. Accordingly, 2-DG significantly increased mitochondrial dysfunction and upregulated Bax/Bcl-2 induced by buforin IIb, increasing cytochrome c release to initiate apoptosis. Moreover, this combination showed low hemolysis and cytotoxicity to normal WPMY-1 cells. Clinical trials of 2-DG have demonstrated the challenges in its use in monotherapy due to poor drug-like characteristics. Many efforts were made to design novel analogs of 2-DG to improve 2-DG’s pharmacokinetics and its drug-like properties [16]. Meantime, more and more preclinical and clinical studies of combined anticancer therapy with 2-DG have been conducted in recent years, and showed a good prospect in anti-cancer therapy. Here, the combination of buforin IIb and 2-DG may provide a universal means by which mitochondria-targeted AMPs may be more potent in combined therapy with 2-DG, which further improves the application of AMPs in cancer treatment. Further studies are needed to develop more mitochondria-targeted AMPs with novel effective 2-DG analogs in cancer therapeutic combination in the future.

## Supplementary Materials

The following are available online. Figure S1: Effects of 2-DG on cell viability in DU145 cells with various concentrations and treatment time. Figure S2: Cell viability of 2-DG toward prostate cancer line PC-3 cells was measured after treatment with 2-DG and/or buforin IIb for 24 h.
