# Supplementary figures and images for: 2-Deoxy-d-glucose Promotes Buforin IIb-Induced Cytotoxicity in Prostate Cancer DU145 Cells and Xenograft Tumors
Source: Molecules. 2020 Dec 7;25(23):5778. doi: 10.3390/molecules25235778 (PMC7730206; doi:10.3390/molecules25235778)

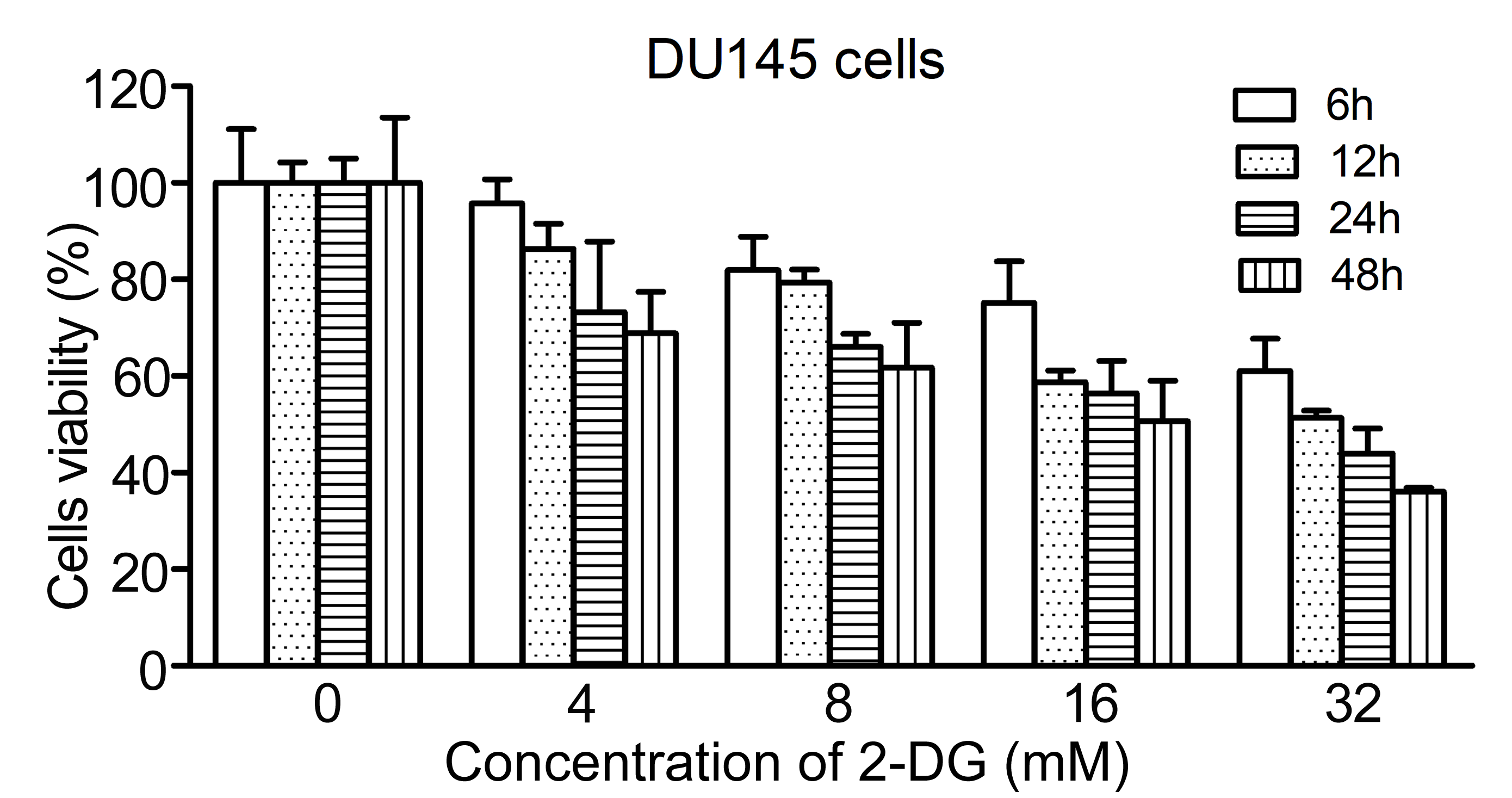

Supplement: Supplementary file 1 [file molecules-25-05778-s001.zip › molecules-979783-supplementary/Supplementary materials/supplementary Figure S1.tif]

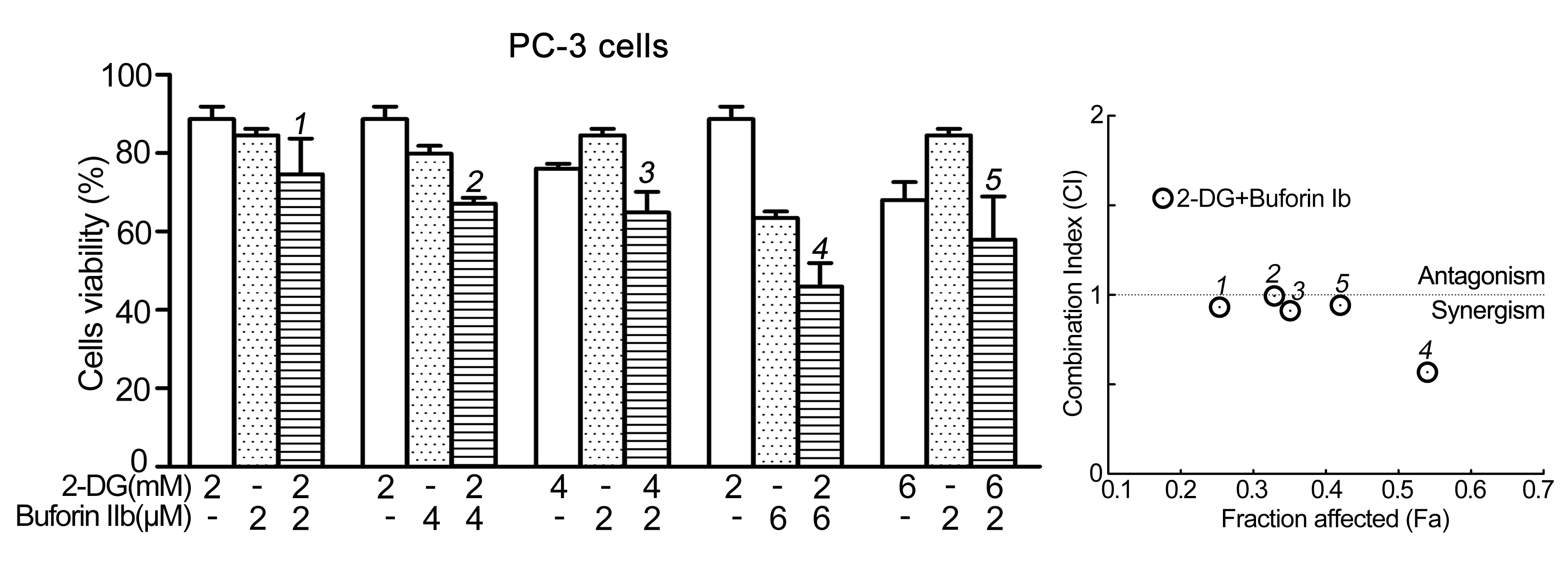

Supplement: Supplementary file 1 [file molecules-25-05778-s001.zip › molecules-979783-supplementary/Supplementary materials/supplementary Figure S2.tif]
