# Supplementary material for: 2-Deoxy-d-glucose Promotes Buforin IIb-Induced Cytotoxicity in Prostate Cancer DU145 Cells and Xenograft Tumors
Source: Molecules. 2020 Dec 7;25(23):5778. doi: 10.3390/molecules25235778 (PMC7730206; doi:10.3390/molecules25235778)
Supplement: Supplementary file 1 [file molecules-25-05778-s001.zip › molecules-979783-supplementary/Supplementary materials/Supplementary materials.doc]

Supplementary Figure S1. Effects of 2-DG on cell viability in DU145 cells with various concentrations and treatment time.

Supplementary Figure S2. Cell viability of 2-DG toward prostate cancer line PC-3 cells was measured after treatment with 2-DG and/or buforin IIb for 24h.
